# Exposure to Traffic-Related Air Pollution in Relation to Progression in Physical Disability among Older Adults

**DOI:** 10.1289/ehp.1510089

**Published:** 2016-03-29

**Authors:** Jennifer Weuve, Joel D. Kaufman, Adam A. Szpiro, Cynthia Curl, Robin C. Puett, Todd Beck, Denis A. Evans, Carlos F. Mendes de Leon

**Affiliations:** 1 Rush Institute for Healthy Aging, Department of Internal Medicine, Rush University Medical Center, Chicago, Illinois, USA; 2 Department of Environmental and Occupational Health Sciences, and; 3 Department of Epidemiology, University of Washington School of Public Health, Seattle, Washington, USA; 4 Department of Medicine, University of Washington, Seattle, Washington, USA; 5 Department of Biostatistics, University of Washington School of Public Health, Seattle, Washington, USA; 6 Department of Community and Environmental Health, College of Health Sciences, Boise State University, Boise, Idaho, USA; 7 Maryland Institute of Applied Environmental Health, School of Public Heath, University of Maryland, College Park, Maryland, USA; 8 Department of Epidemiology, University of Michigan School of Public Health, Ann Arbor, Michigan, USA

## Abstract

**Background:**

Physical disability is common though not inevitable in older age and has direct bearing on a person’s ability to perform activities essential for self-care and independent living. Air pollution appears to increase the risk of several chronic diseases that contribute to the progression of disability.

**Objective:**

We evaluated long-term exposure to traffic-related air pollution (TRAP) in relation to progression in physical disability.

**Methods:**

We conducted our investigation within the Chicago Health and Aging Project. We measured participants’ exposures to TRAP using two surrogates: residential proximity to major roads (1993 onwards) and ambient concentrations of oxides of nitrogen (NOX; 1999 onwards), predicted via a geographic information systems-based spatiotemporal smoothing model (cross-validation R2 = 0.87) that incorporated community-based monitoring and resolved intraurban exposure gradients at a spatial scale of tens of meters. Participants’ lower-extremity physical ability was assessed every 3 years (1993–2012) via tandem stand, chair stand, and timed walking speed.

**Results:**

In multivariable-adjusted analyses (n = 5,708), higher long-term NOX exposure was associated with significantly faster progression in disability. Compared with the 5-year decline in physical ability score among participants in the lowest quartile of NOX exposure, decline among those in the highest exposure quartile was 1.14 units greater (95% confidence interval [CI]: –1.86, –0.42), equivalent to 3 additional years of decline among those in the lowest exposure quartile. The association was linear across the continuum of NOX exposure: per 10-ppb increment in exposure, the 5-year decline in physical ability score was 0.87 unit greater (95% CI: –1.35, –0.39). Proximity to a major road was not associated with disability progression (n = 9,994).

**Conclusions:**

These data join a growing body of evidence suggesting that TRAP exposures may accelerate aging-related declines in health.

**Citation:**

Weuve J, Kaufman JD, Szpiro AA, Curl C, Puett RC, Beck T, Evans DA, Mendes de Leon CF. 2016. Exposure to traffic-related air pollution in relation to progression in physical disability among older adults. Environ Health Perspect 124:1000–1008; http://dx.doi.org/10.1289/ehp.1510089

## Introduction

Aging-related physical disability has become a critical health concern because of its high associated health care costs and because of the rapidly growing number of older Americans (Administration on Aging 2009; Federal Interagency Forum on Aging-Related Statistics 2010). Disability represents the combined impact of common, often comorbid chronic diseases and subclinical pathologic processes on a person’s ability to perform tasks and activities that are essential for self-care and independent living. Regarded as a key indicator of overall health in older adulthood (Manton et al. 1997; Pope and Tarlov 1991), disability becomes increasingly prevalent with advancing age, from ~10% among 65- to 74-year-olds in the United States to > 50% of those > 85 years old (Manton et al. 2006; Seeman et al. 2010). Disability is the primary reason for the requirement of long-term care (Kemper 1992; McKinlay et al. 1995) and accounted for $350 billion in Medicare costs in 2009 (Manton et al. 2007). Therefore, reducing the number of disability-affected years during late life is of interest because disability threatens an individual’s independence and increases both health care utilization and the burden on informal caregivers (Fries 2003).

Exposure to air pollution—particularly long-term exposure—appears to have multiple adverse health effects, including increased risks for cardiovascular and respiratory disease [Brook et al. 2010; Brunekreef et al. 2009; Committee on the Medical Effects of Air Pollutants 2006; Health Effects Institute (HEI) 2010; Pope and Dockery 2006], stroke (Brook et al. 2010; Pope and Dockery 2006; Wellenius et al. 2012b), age-related cognitive decline (Tonne et al. 2014; Weuve et al. 2012a), rheumatoid arthritis (Hart et al. 2009), and diabetes (Andersen et al. 2012; Brook et al. 2010; Chen et al. 2013; Coogan et al. 2012; Krämer et al. 2010; Pearson et al. 2010). High exposures also appear to be associated with pathophysiologic processes that contribute to these conditions (e.g., hypertension, systemic inflammation) (Brook et al. 2010; HEI 2010; Pope and Dockery 2006; Wellenius et al. 2013). Some of these health effects may be more pronounced in older adults (Fischer et al. 2003; Goldberg et al. 2000; Medina-Ramón and Schwartz 2008) in part because of the interplay between exposures and chronic disease processes (Anderson et al. 1990; Annesi-Maesano et al. 2003; Segal et al. 2002). Given that physical disability is a common functional consequence of these subclinical processes and chronic disease conditions (CDC 2009; Black and Rush 2002), it is likely that exposure to air pollution also influences physical disability at advanced ages.

We studied the relationship between long-term exposure to air pollution and the progression of physical disability in older age by combining data from participants in the Chicago Health and Aging Project (CHAP) and exposure estimates derived using a model developed for the Multi-Ethnic Study of Atherosclerosis and Air Pollution (MESA Air) Study. Our work focused on traffic-related air pollution (TRAP), a major source of exposures to toxic pollutants in urban settings that include but are not limited to nitrogen oxides, carbon monoxide, ozone, and ultrafine suspended particles (Adar and Kaufman 2007; HEI 2010). We hypothesized that physical disability would progress faster among those with greater long-term exposure to TRAP. An increasing number of people live in urban areas (United Nations, Department of Economic and Social Affairs, Population Division 2014), where TRAP is highly concentrated but is not uniformly distributed (Jerrett et al. 2005), providing impetus for determining whether TRAP exposure influences the disabling process and for studying this question in intra-urban settings.

## Methods

### Study Population

We conducted our investigation within CHAP, a longitudinal study of residents of a geographically defined area on the south side of Chicago, IL, who were aged 65 and older (Bienias et al. 2003; Evans et al. 2003). From 1993 to 1996, an original cohort of 6,157 participants was recruited into CHAP (79% of all eligible persons, established by community census); 4,644 newly age-eligible participants were recruited in successive cohorts. Until 2003, participants were drawn from three contiguous neighborhoods. Starting in 2003, participants were also recruited from an adjoining neighborhood. Altogether, the study area is approximately 15 mi^2^ (39 km^2^) with participants living throughout. All participants underwent triennial in-home assessments during which they completed questionnaires and underwent evaluation of their cognitive and physical function (see “Assessment of Physical Disability”); 85% of all survivors, on average, completed follow-up visits after their baseline evaluations.

### Assessment of Exposure to Traffic-Related Air Pollution

We estimated participants’ exposures to TRAP using two surrogates: residential proximity to major roads and outdoor concentrations of nitrogen oxides (NO*_X_*) at each participant’s residential location, as predicted by the spatiotemporal model described below. Participants’ exposure measurements were assessed specific to the time periods during which they resided in those locations. These measurements depended on participants’ residential locations and the periods during which they resided in those locations. All available residential locations were geocoded in ArcGIS 9.3 [Environmental Systems Research Institute (ESRI), Inc.] using the Tele Atlas® Dynamap® 2000 v.16.1 road network (Boston, MA). Geocodes were calculated using side offsets of 30 ft. Automated geocoding was used for all addresses with an 80% or greater match on both spelling sensitivity and match score. Addresses that could not be automatically matched with at least 80% accuracy were matched manually. Nearly all of the locations (98%) were sufficiently complete and correct to allow geocoding to an exact location.

**Figure 1:**
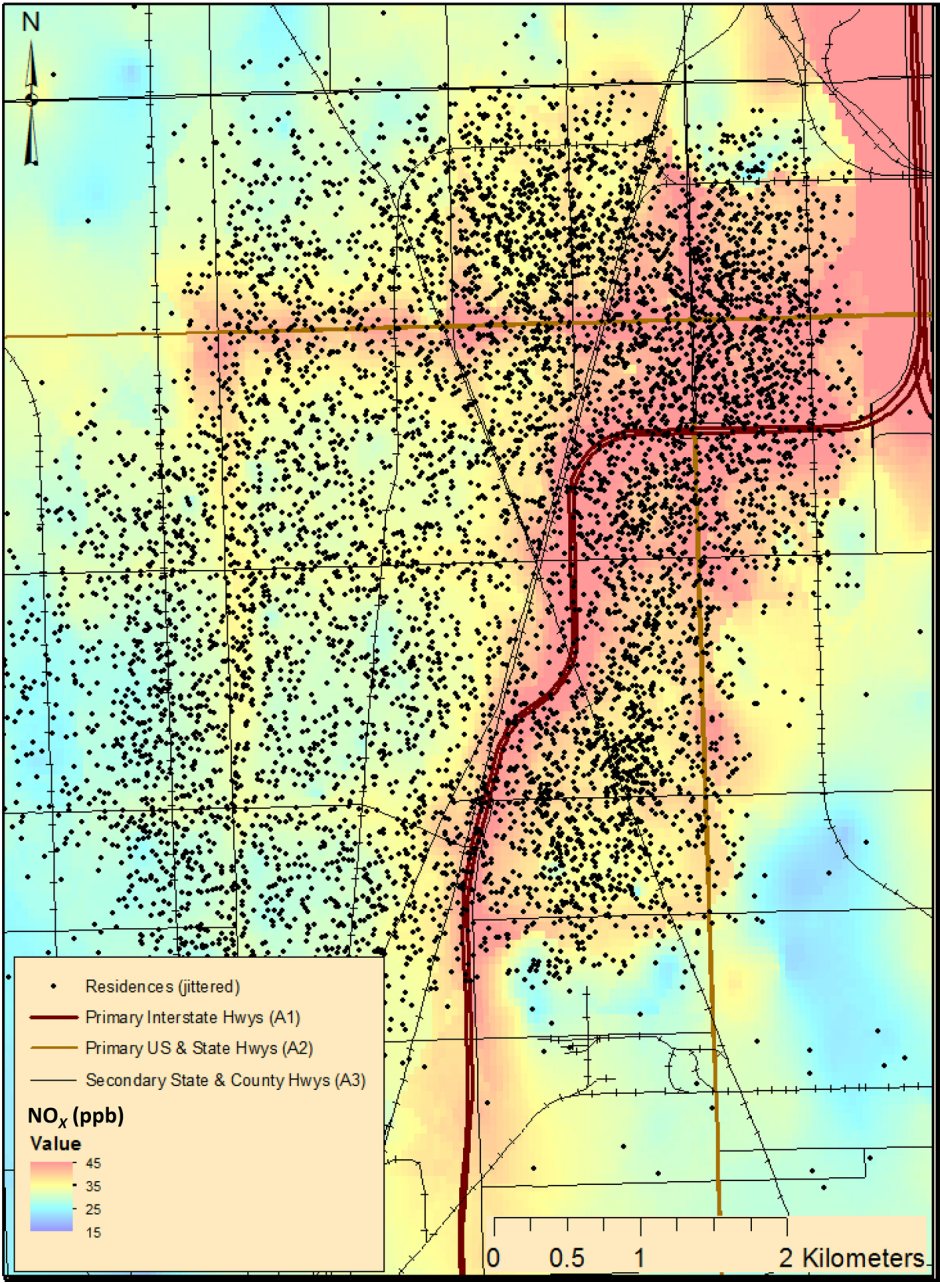
Average concentrations of oxides of nitrogen (NO*_X_*), in parts per billion, major roadways in the Chicago Health and Aging Project (CHAP) study area (1999–2011), and participants’ residential locations. Residential locations have been moved slightly (jittered) to protect participants’ confidentiality.

***Exposure to ambient NO*_X_.** Our primary measure of exposure was local outdoor concentrations of NO*_x_* which serve as tracers of TRAP. For each CHAP residential location and for each 2-week interval between 1999 and 2011 (e.g., 1–14 January 1999; 15–28 January 1999; etc.), we generated predicted outdoor, ambient-source NO*_x_* concentration using spatiotemporal modeling optimized via maximum likelihood methods that were developed by MESA Air investigators (Keller et al. 2015; Sampson et al. 2011). These investigators developed a geographic information systems-based model for Chicago (Gill et al. 2011) that incorporated hundreds of variables including geographic features, roadway information, and data from a community-monitoring campaign. They calculated a suite of geographic covariates for all MESA residential locations in Chicago. These covariates included distances to a variety of features (nearest airport, railroad, rail yard, city center, major roadway, roadway intersection, port, air pollution emission source, and truck route), information about the local geography within buffers of various sizes (sum of roadway lengths; sum of truck route roadway lengths; counts of intersections; percentages of residential, commercial, and industrial land use; percentage of impervious surface cover; percentage of vegetative land cover; population density; and total air pollution emissions). Additional geographically based covariates included absolute and relative elevation, urban topography (to identify features such as “street canyons”), traffic dispersion model outputs (i.e., CALINE), and a variety of census data. The model also incorporated data from a community-monitoring campaign and included a long-term spatial mean, temporal trends with spatially varying coefficients, and a spatiotemporal residual. The mean structure was derived from the large set of geographic covariates that was reduced using partial least squares regression. Temporal trends were estimated from observed time series, and spatial smoothing methods were used to borrow strength between observations. The resulting model had a 10-fold cross-validation *R* ^2^ of 0.87 (described in detail by Keller et al. 2015). The model was finely resolved both temporally (with predictions specific to 2-week intervals) and spatially (on the order of tens of meters), allowing it to predict temporally varying intraurban exposure gradients. Figure 1 shows average predicted NO*_X_* concentrations from 1999 to 2011 in the CHAP study area, which was fully contained by the MESA Air modeling area.

The location-specific 2-week NO*_x_* concentrations formed the building blocks for estimating a given CHAP participant’s long-term NO*_x_* exposure over a specific interval, as described in “Statistical Analyses.” [Although the MESA Air Study developed Chicago-based models for other ambient pollutants, including fine and coarse particulate matter (PM) (Keller et al. 2015; Zhang et al. 2014), the estimated concentrations did not vary sufficiently within the CHAP area or were unavailable for CHAP participants at the time of the present study.]

***Distance to major roads.*** We conducted secondary analyses using residential distance to major roads. Although predicted NO*_X_* concentration is a more refined measure of TRAP exposure, the sample with road proximity estimates included 75% more participants. For each residential location, we estimated distances to different classes of road: census feature classes A1 (interstate expressways), A2 (generally state highways), and A3 (some state highways and county roads), as well as truck routes, which are a major source of exposure to diesel particles.

### Assessment of Physical Disability

Every 3 years, participants underwent performance-based assessments of their basic lower-extremity functions. The Short Physical Performance Battery (SPPB) measures the participant’s balance, lower-extremity strength, and gait. It entails three progressively more difficult standing tests [standing with feet side by side, a semi-tandem stand (one foot adjacent to but a half foot in front of the other), and then a full tandem stand (one foot immediately in front of the other)], each for up to 10 sec; five repeated chair stands (rising from chair to standing position); and a timed walking test over an 8-ft course (gait speed). The SPPB has been used in a variety of epidemiologic studies of aging and has well-established reliability and validity (Freiberger et al. 2012; Guralnik et al. 1994, 2000; Mendes de Leon et al. 2002, 2005; Ostir et al. 2007). We divided scores on each of the three component tests into quintiles of performance (using cutoffs from baseline measurements), establishing an additional category for those who were unable to complete the test. Thus, scores ranged from 0 (inability to complete the test) to 5, which we summed across the three tests for a summary measure of physical function (range, 0–15). Higher scores indicated a higher level of physical ability.

### Statistical Analyses

***Measures of exposure.*** Our analyses targeted measures that reflected long-term TRAP exposure that occurred prospectively with respect to disability status.

To estimate each participant’s long-term exposure to NO*_X_*, we averaged the predicted NO*_X_* concentrations at each of her/his residential locations over the 5-year period preceding the first eligible physical performance assessment. Because the NO*_X_* model began in 1999 and both enrollment in CHAP and follow-up assessments were ongoing, we identified the first physical performance assessment and the NO*_X_* averaging period to use for these analyses following an iterative process. First, we designated a date that would be an initial placeholder for starting the NO*_X_* averaging. 1 January 1999 was the working start date for participants who were previously enrolled in CHAP or who enrolled by 1 January 2001 (2 years later), corresponding to the assumption that most noninstitutionalized participants did not move between 3-year study cycles. For the remaining participants, we used responses to a question posed in the third field cycle (2000–2002) that asked how many years they had lived in their current residential location, subtracting the number of years reported from their enrollment dates. The resulting date became the working start date, unless it fell before 1 January 1999, in which case, the latter was designated as the working start date. The working start date for all others (those who enrolled after 1 January 2001 and who did not respond to the questionnaire) was their enrollment date minus 2 years. As warranted, the 5-year NO*_X_* average accounted for multiple locations and time residing at those locations.

From each participant’s working start date, we identified the first physical performance assessment that occurred 5 or more years later. Data from this and all subsequent assessments were used in our analyses. We then recalibrated each participant’s NO*_X_* averaging period, designating the period as the 5 years before this physical performance assessment. For nearly all of these participants (94%), NO*_X_* exposure averaging began in 1999. We excluded participants who moved to a nursing home within 5 years following their averaging start date. We analyzed this measure in relation to concurrent and subsequently assessed physical performance (through 2012), analyses that included 10,911 observations from 5,708 participants.

We categorized distances to each road type as ≥ 200 m, 100–199 m, 50–99 m, and < 50 m (Karner et al. 2010); we also generated a composite measure equal to the minimum of the distances to truck routes and class A1 and A2 roads. We analyzed baseline roadway proximity (1993–2000) in relation to concurrent and to subsequently assessed physical performance (1993–2012). These analyses included 23,434 observations from 9,994 participants.

***Association of TRAP exposure with physical disability.*** We evaluated the relationship of TRAP exposure with physical disability and disability progression by fitting multivariable-adjusted generalized estimating equation (GEE) regression models, with identity links (Zeger and Liang 1986), of repeated physical performance score. We fitted separate models for each measure of exposure, beginning with categories of the measure (for long-term NO*_X_* exposure, quartiles) and progressing to continuous measures as we established linearity in the association between exposure and physical performance. The GEE approach accounted for the correlation of within-individual repeated physical performance scores over the cycles.

All models included terms for age at the baseline physical performance assessment (continuous); sex; race (African American, white); years of education (continuous); household income (three categories and missing); baseline smoking status (current, former, never); time, in years, since the baseline physical performance assessment (continuous); and the cross-products of time with age, sex, race, education, income (distance to road only), and the air pollution exposure variable. Our principal interest was in the estimated parameter corresponding to the time-exposure cross-product, directly interpreted as the difference in the annual rate of change in the physical performance score across levels of exposure. For reporting, we converted the parameter estimate into the difference in score change over a 5-year period. The results remained unchanged with further adjustment for other factors (e.g., alcohol consumption) and cross-products of other covariates with time (data not shown). Because baseline disability and health status was likely to be a result of TRAP exposure rather than a cause of it, we did not adjust our analyses for these factors. Tests for linear trends across categories of exposure were conducted using a single term taking on the median values of each category. We tested subgroup differences in the association of exposure with decline in physical performance using three-way cross-products of the exposure measure, time, and group indicator.

***Accounting for differential attrition.*** Attrition, particularly from mortality, is common in longitudinal studies of older adults, and participants bearing greater physical disability have an increased likelihood of being lost to follow-up (Chatfield et al. 2005; Matthews et al. 2006). If the risk factor of interest also influences attrition, the resulting differential attrition can bias estimates of the causal association between that factor and progression in disability (Hernán et al. 2004; Weuve et al. 2012b). To explore the relationship of air pollution exposure and physical performance to attrition in our data, we fitted pooled logistic regression models of continuation (the inverse of attrition) from study cycle to study cycle, following a procedure that we previously adopted and refined (Tchetgen Tchetgen et al. 2012; Weuve et al. 2012b). We fitted separate models for each of the distance to roadway and NO*_X_* measures and included a variety of time-invariant (e.g., baseline age, sex, race, education) and time-varying terms (e.g., cognitive function score, diabetes), along with the exposure of interest and a time-varying term for physical performance (i.e., score at most recent visit). Notably, these continuation models can include variables that may be plausible intermediates on the causal pathway between air pollution exposure and disability. By contrast, it would not be appropriate to include these plausible intermediate variables in models estimating the total effect of air pollution exposure on disability progression.

Of the CHAP participants in our analyses of NO*_X_* exposure, 24% died and 12% dropped out at some point after their index physical disability assessment. The risk of attrition increased with higher long-term NO*_X_* exposure [hazard ratio (HR) per 10 ppb increment, 1.14; 95% confidence interval (CI): 0.99, 1.31] and lower previous physical performance score [HR per standard deviation (sd) in score, 0.79; 95% CI: 0.74, 0.85]. (Roadway proximity was not associated with attrition.) Therefore, for analyses of NO*_X_* exposure, we used inverse probability-of-continuation-weighted regression to correct, at least in part, the possible bias introduced by the attrition related to this exposure and outcome (Cole et al. 2005; Hernán et al. 2000; Tchetgen Tchetgen et al. 2012; Weuve et al. 2012b). For each observation in our GEE model of NO*_X_* exposure and physical performance, we computed stabilized weights, as previously described (Weuve et al. 2012b), as the inverse of the predicted probability of continuation multiplied by the predicted probability of continuation from a model containing only the time-invariant variables of the GEE model.

***Sensitivity analyses.*** We performed several analyses to assess the robustness of our findings. For NO*_X_*, these included restricting analyses to participants who did not move over the course of the exposure averaging period; exploring the potential influence of very small or large continuation weights on the results by using weights truncated at the 0.5th and 99.5th percentiles of the weight distribution; and repeating analyses using NO*_X_* averages computed over 1, 2, 3, and 4 years. Averaging exposures over the 5 years before disability more accurately characterizes the historic long-term exposures hypothesized to affect the course of physical disability than does averaging over shorter time periods. However, this approach limits the amount of disability data included in the analyses: the number of observations and participants included in these alternative analyses ranged from 12,097 observations from 6,041 participants (4-year NO*_X_* average) to 16,479 observations from 7,291 participants (1-year NO*_X_* average).

Sensitivity analyses applied to distance to road included exploring the alternative A3 road proximity categories ≥ 500 m, 251–499 m, 101–250 m, and ≤ 100 m (Karner et al. 2010; Wellenius et al. 2012a); analyzing the composite variable, in which we classified participants as living “near a major road” if their residence was < 100 m from an A1 or A2 road, < 100 m from a truck route, or < 50 m from an A3 road; and restricting analyses to the subgroup of participants who were in the analyses of 5-year NO*_X_* exposure.

Sensitivity analyses of both TRAP measures included differences in association by baseline smoking status, race, and adjustment for a composite measure of area-based socioeconomic status (SES) (see Supplemental Material, “Computation of area-level socioeconomic status”).

All CHAP participants provided their written informed consent to engage in the CHAP protocols. This study was approved by the Institutional Review Boards of Rush University Medical Center and the University of Washington.

## Results

### Exposure to NO*_X_*

Long-term exposure to NO*_X_*, as indicated by predicted concentrations averaged over 5 years, ranged from 20.7 to 56.0 ppb, with a mean of 39.7 ppb (standard deviation, 5.8 ppb). NO*_X_* exposure was markedly higher, on average, among participants who were African American (versus white), and who had less formal education and lower household incomes (Table 1). Participants living in areas with higher NO*_X_* concentrations were also less likely to rate their health as excellent.

**Table 1:** Participant characteristics,*^a^* by quartile of long-term exposure to oxides of nitrogen.

Characteristic	Overall^*c*^ *n* = 5,708	Quartile of long-term^*b*^ NO_*X*_ exposure				*p*-Value
		Lowest (20.7–36.2 ppb) *n* = 1,431	Second (36.3–39.6 ppb) *n* = 1,435	Third (39.7–43.7 ppb) *n* = 1,423	Highest (43.8–56.0 ppb) *n* = 1,419	
Age, years (mean ± SD)	75.9 ± 7.1	76.8 ± 8.0	76.2 ± 7.2	75.4 ± 6.8	75.2 ± 6.3	< 0.0001
Male, %	2,071 (36)	36	39	34	37	0.05
Race, %						< 0.0001
African American	3,668 (64)	35	41	83	98
White	2,040 (36)	65	59	17	2
Education, years (mean ± SD)	12.8 ± 3.2	14.0 ± 3.4	13.1 ± 3.1	12.1 ± 3.1	11.9 ± 3.3	< 0.0001
Household income, %^*d*^						< 0.0001
< 15,000 USD	1,149 (20)	13	15	26	27
15,000–29,999 USD	2,129 (37)	26	33	43	48
≥ 30,000 USD	2,310 (40)	59	50	29	24
Missing	120 (2)	3	2	2	1
Self-rated health, %^*d*^						< 0.0001
Excellent	1,201 (21)	28	23	18	15
Good	2,828 (50)	50	51	48	50
Fair	1,400 (25)	19	21	29	29
Poor	275 (5)	4	5	6	5
Global cognitive score, standard units (mean ± SD)	0.3 ± 0.8	0.4 ± 0.7	0.4 ± 0.7	0.2 ± 0.8	0.2 ± 0.8	< 0.0001
Social network score, median (iqr)^*e*^	6 (7)	6 (8)	6 (7)	6 (6)	6 (6)	< 0.0001
Walks > 3 hr per week, %	1,030 (18)	19	20	16	17	0.01
Smoking status, %^*d*^						0.004
Never	2,673 (47)	48	46	47	46
Former	2,460 (43)	44	45	41	42
Current	575 (10)	8	9	12	11
Alcohol intake, %^*d*^						< 0.0001
None	3,693 (65)	56	60	70	72
Moderate	1,698 (30)	36	33	26	24
Heavy	315 (6)	8	7	4	4
Systolic blood pressure, mmHg (mean ± SD^*e*^)	135 ± 20	134 ± 21	135 ± 19	136 ± 19	136 ± 19	0.2
Diastolic blood pressure, mmHg (mean ± SD^*e*^)	77 ± 11	78 ± 12	76 ± 11	78 ± 11	77 ± 11	0.005
Self-reported history of disease
Hypertension, %	3,905 (68)	65	62	73	73	< 0.0001
Cardiovascular disease, %	818 (14)	14	15	15	14	0.6
Stroke, %	648 (11)	9	11	13	12	0.007
Cancer, %	1,308 (23)	28	23	22	19	< 0.0001
Minimum distance to Class A3 road, %^*d*^						< 0.0001
≤ 50 m	640 (11)	5	8	15	16
50 m–99 m	601 (11)	7	10	14	12
100 m–199 m	1,372 (24)	24	26	24	22
≥ 200 m	2,095 (54)	64	56	47	49
Near busy road^*f*^, %	1,018 (18)	8	11	20	33	< 0.0001
Moved over the course of follow-up, %	252 (4)	5	5	4	3	0.01
Physical performance score (mean ± SD)	9.5 ± 4.1	9.8 ± 4.1	10.0 ± 4.1	9.1 ± 4.1	9.0 ± 4.0
Abbreviations: iqr, interquartile range; NO_X_, oxides of nitrogen; SD, standard deviation; USD, U.S. dollars. ^***a***^Unless specified, all characteristics reflect baseline values, that is, the values at the first assessment used in analyses of 5-year average NO_X_ exposure. ^***b***^Predicted exposures averaged over 5 years. ^***c***^Where percentages are listed in the “Overall” column, counts are also provided [*n* (%)]. ^***d***^Some columns do not sum to 100% because of rounding. ^***e***^Data on self-rated health, social network score, walking, alcohol intake, blood pressure, and self-reported history of the four chronic conditions were missing for a small percentage of participants (< 2%). Values shown reflect non-missing responses. Data on these characteristics were not used in the association models. ^***f***^Near busy road: < 100 m from A1, A2, or truck route, or < 50 m from A3.						

Unless otherwise specified, all NO*_X_* results presented below are from weighted analyses. In unadjusted analyses, irrespective of weighting, higher long-term NO*_X_* exposure was associated with worse physical performance at baseline (Table 1; see Figure S1 left). However, this pattern was reversed with multivariable adjustment. For example, baseline physical performance scores were, on average, 0.26 unit higher per 10-ppb increment in NO*_X_* exposure (95% CI: 0.06, 0.45). Nonetheless, the higher baseline scores associated with NO*_X_* exposure were eclipsed over time by the faster rate of decline in performance among those with higher exposures (see Figure S2). Specifically, decline in physical performance was significantly more rapid among participants exposed to higher long-term concentrations of NO*_X_* (*p*_trend_ = 0.002; Table 2; see Figure S2). Compared with participants in the lowest exposure quartile, performance among participants in the highest exposure quartile declined by 1.14 units (95% CI: –1.86, –0.42) more per 5-year interval. With NO*_X_* exposure modeled as a continuous variable, a 10-ppb increment in exposure corresponded to a 0.87-unit excess decline in performance (95% CI: –1.35, –0.39). Placing the magnitude of these estimates in context, those in the highest NO*_X_* quartile declined in performance over a 5-year interval to the same degree that those in the lowest quartile declined over an 8-year interval. Likewise, comparing participants 10 ppb apart in exposure, the 5-year decline in physical performance in the more highly exposed individuals was equivalent to the expected 7-year decline in the less-exposed individuals.

**Table 2:** Adjusted*^a^* differences [and 95% confidence interval (CI)] in 5-year change in physical performance by exposure to traffic-related air pollution.

Exposure measure	Difference in 5-year change in physical performance score (95% CI)	
Long-term exposure to oxides of nitrogen (NO_*X*_)^*b*^ (*n* = 5,708)**
Quartile of long-term NO_*X*_ exposure
Lowest (20.7–36.2 ppb)	0.00	Reference
2nd (36.3–39.6 ppb)	–0.49	(–1.06, 0.08)
3rd (39.7–43.7 ppb)	–0.52	(–1.13, 0.10)
Highest (43.8–56.0 ppb)	–1.14	(–1.86, –0.42)
		*p*_trend_^*c*^ = 0.002
Per 10-ppb increment in long-term NO_*X*_ exposure	–0.87	(–1.35, –0.39)
Residential distance to road (*n* = 9,994)
Minimum distance to nearest truck route or A1 or A2 road
≥ 200 m	0.00	Reference
100–199 m	0.20	(0.02, 0.38)
50–99 m	–0.02	(–0.28, 0.23)
< 50 m	–0.17	(–0.62, 0.28)
		*p*_trend_^*c*^ = 0.2
Distance to nearest A3 road
≥ 200 m	0.00	Reference
100–199 m	–0.24	(–0.38, –0.10)
50–99 m	–0.12	(–0.32, 0.08)
< 50 m	–0.04	(–0.25, 0.16)
		*p*_trend_^*c*^ = 0.02
NO_X_, oxides of nitrogen ^***a***^Adjusted for age, sex, race, education, income, and smoking status. ^***b***^Predicted exposures averaged over 5 years. ^***c***^Trend *p*-values computed from models containing a term that took on the median values of each category.		

**Figure 2:**
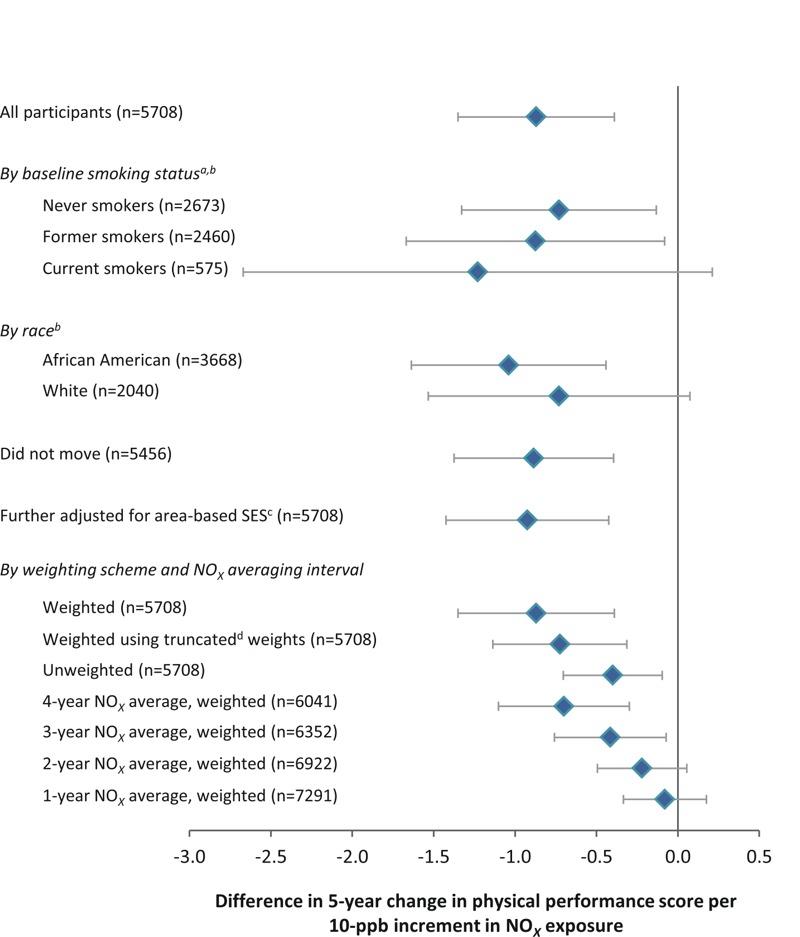
Adjusted difference in 5-year change in physical performance score per 10-ppb increment in long-term exposure to oxides of nitrogen (NO*_X_*): subgroup and sensitivity analyses. Estimates are adjusted for age, sex, race (as appropriate), education, income, and smoking status. Predicted exposures are averaged over 5 years, unless another interval is specified. *^a^*Smoking status for each participant is defined at the time of the first physical performance assessment used in the analyses of long-term NO*_X_* exposure. *^b^*Interaction *p*-values for differences in the NO*_X_*-performance change association by smoking and race are 0.7 and 0.5, respectively. *^c^*Area-based socioeconomic status (SES) refers to the composite area-based measure of SES. *^d^*Truncated weights refer to analyses with extreme weights truncated to the lowest 0.5 percentile and highest 99.5 percentile.

***Sensitivity analyses.*** Results were smaller in magnitude when we truncated the analytical weights (Figure 2; see Table S1). Unweighted results were smaller still but remained statistically significant. From analyses in which we averaged NO*_X_* exposures over 4 years and in which the earliest disability assessment included was in 2003, the results were consistent with the results from analyses of 5-year average NO*_X_* exposures. Estimated associations using shorter averaging intervals were discernibly smaller in magnitude and, with respect to 2- and 1-year exposure averages, were not statistically significant. Results among participants who did not move and results adjusted for the composite measure of area-level SES were similar to those from the primary analyses. The results did not vary substantially by race or by smoking status (Figure 2).

### Distance to Road

Residential distances to road types were distributed most extensively across categories of A3 road (see Table S2): approximately half of the participants lived < 200 m from the nearest A3 road, and 9% overall lived < 50 m away. Participants who lived closer to major roads, including A3 roads, were more likely to be African American (e.g., 73% of those < 50 m from an A3 road were African American, compared with 61% of those living ≥ 200 m from an A3 road) and of lower socioeconomic status, indicated by fewer years of education and lower household income (see Table S3).

Compared with ≥ 200 m to A3 road, shorter distance was associated with significantly worse physical performance at baseline in both unadjusted (see Table S3) and multivariable-adjusted analyses (see Figure S3; *p*_trend_ = 0.004). With proximity to A3 roadway < 200 m, performance declined more rapidly over time (Table 2), but these findings were small in magnitude. Moreover, although the linear trend *p*-value was 0.02, those living at 100- to 199-m distances declined an excess 0.24 unit (95% CI: –0.38, –0.10) more in performance over 5 years than those living at ≥ 200 m distances, whereas those living at < 50 m distances declined at nearly the same rate as those living at ≥ 200 m distances. Minimum distance to truck route/A1 road/A2 road was not consistently associated with baseline physical performance (results not shown) or longitudinal deterioration in performance (Table 2).

***Sensitivity analyses.*** The results were similar under the alternative scheme for classifying distance to A3 road, with closer proximity associated with significantly worse physical performance at baseline (see Figure S3; *p*_trend_ = 0.01). However, proximity to A3 road, using these alternative categories, was not associated with longitudinal decline in performance (see Table S1). As of enrollment, 16% of participants lived “near a busy road” (see Table S2). This status was not associated with baseline physical performance (results not shown) or with longitudinal decline in performance (see Table S1).

Restricting analyses to the 5,708 participants who were in the analyses of 5-year NO*_X_* exposure, we analyzed residential distance to A3 road at the time at which their 5-year NO*_X_* averaging began (1999 for most). Compared with the primary analyses, these analyses revealed a less-consistent association of distance to A3 roadway with baseline physical disability, but a clearer gradient in disability progression was observed with closer proximity to A3 road (*p*_trend_ = 0.05; see Table S1). These results are consistent with the findings on NO*_X_* exposure in the same population.

The results did not vary significantly by smoking status or race (results not shown). Further adjustment for area-based SES modestly attenuated the association of distance to A3 roadway with baseline physical performance (results not shown).

## Discussion

In this large study of urban-dwelling older adults, physical disability progressed more rapidly among those with higher long-term exposure to ambient NO*_X_*, a measure of exposure to TRAP. Comorbid conditions are extremely common among older adults, and studies of air pollution and single-system endpoints, such as cardiovascular and respiratory disease, may overlook the aggregate effects of air pollution exposure on multiple organ systems in this population. Evidence supporting such a relationship could motivate the use of exposure-reduction policies to reduce the burden of physical disability on the population, particularly among African Americans, who, on average, experience higher air pollutant exposures than do whites (Lopez 2002). Our study assessed physical disabilities that predict an individual’s subsequent independence. In contrast to performance-based assessments of physical disability, activities-of-daily-living (ADL) disability entails self-reported difficulty in performing specific tasks. Factors other than physical disability, such as depressive symptoms and cognitive status, can influence ADL reporting. Moreover, performance-based assessments can detect lower-extremity deficits even in persons without an ADL disability (den Ouden et al. 2011). This subclinical detection capacity is relevant to characterizing the full range of air pollution’s effects on the health of older adults: persons with chronic health conditions that are associated with air pollution exposure [e.g., lung and heart disease (Eisner et al. 2008; Khan et al. 2013)] perform substantially worse on these tests than those without such chronic conditions, and comorbidities worsen performance further (see, for example, Cesari et al. 2006). However, to a more subtle but nevertheless detectable extent, performance is also worse among persons who are free of clinical disease but who have indications of subclinical pathologies, such as inflammation or atherosclerosis (Brinkley et al. 2009; den Ouden et al. 2014).

Our study is, to our knowledge, the first to prospectively evaluate the relationship between long-term exposure to air pollution and progression in physical disability. Previous epidemiologic studies of air pollution exposure and disability were cross-sectional, evaluated acute effects of short-term exposures, used single self-reported measures of disability and exposures measured at the level of the community, or did not focus on older adults. In cross-sectional data from adults representative of the U.S. population in 1976–1981, higher exposure to ambient fine PM, estimated at the metropolitan level, was associated with more restricted activity days among adults 18–65 years of age (Ostro 1987). Similar findings emerged from a time series study of adults (mean age, 43 years) in Toronto, Canada, from 1994 to 1999, when overall pollutant exposures were much lower than in the period studied in the Ostro (1987) study: higher levels of short-term exposure to fine PM and carbon monoxide were both associated with increased disability days (Stieb et al. 2002). The only previous study among older adults was set in China, where high community-level ambient air pollution was associated with having at least one ADL disability 7 years after the period during which the exposure was assessed (Zeng et al. 2010).

Our study introduces several critical strengths to advance the field, notably: we provide individual-level estimates of long-term exposure and performance-based assessments of the trajectory of physical disability, and we account for possible bias from selective attrition. However, several limitations merit attention. First, as necessitated by research on the health effects of exposure to long-term air pollution, our study follows an observational design, making it susceptible to bias from confounding. Confounding by socioeconomic disadvantage is a particular concern because indicators such as education and income track inversely with exposure. The CHAP population is well characterized in this regard, and we took several approaches to adjust for socioeconomic disadvantage, including the use of cross-product terms between several socioeconomic indices and time. In sensitivity analyses, further adjustment for area-based socioeconomic disadvantage did not attenuate the association of NO*_X_* exposure with decline in physical performance (Figure 2). Finally, compared with unadjusted and unweighted results, weighted multivariable-adjusted results actually indicated a more deleterious association of long-term NO*_X_* exposure with this outcome (see Figure S1).

Second, the exposure measurements were based on participants’ residential locations and thus were limited by the assumption that participants spent most of their time at home, although this assumption may be reasonable for older individuals (Spalt et al. 2015). Personal air monitoring devices are impractical for long-term exposures in large epidemiologic studies. By contrast, GIS-based spatiotemporal models offer the ability to estimate individual exposures for thousands of participants, customized to time period. The MESA Air NO*_X_* exposure model, developed specifically for Chicago, accounts for small-scale variations in exposure from traffic and nontraffic sources, allowing us to distinguish among different exposure levels over space and time within the study area (Cohen et al. 2009; Keller et al. 2015; Sampson et al. 2011). We took several steps toward making the NO*_X_* measures optimal reflections of the long-term exposures that might influence disability. We first averaged predicted exposures over the 5 years before the disability assessment, offering gains in precision and accuracy beyond the use of exposure measures averaged over shorter periods or periods encompassing disability follow-up. Next, to avoid reverse causation bias, we excluded participants living in nursing homes. In such cases, estimated exposures could result from, rather than lead to, disability.

Third, the use of inverse probability-of-continuation weights addressed selection bias in the estimates of change in physical performance, but it had no bearing on bias from differential selection into the study and by extension, no bearing on estimates of baseline differences in physical performance. Assuming that selection into the study fell under the same influences as continuation in the study, estimated associations of TRAP exposure with baseline physical performance would be biased upward (even in the presence of no relationship), possibly explaining the observation of better baseline function with higher NO*_X_* exposure.

The extent to which our results might generalize to other settings is unclear. For example, the study population comprises African American and white older residents of a large city in the midwestern United States. Coexposures and other factors that may influence susceptibility to TRAP’s effects on disability may be present (or lacking) in other populations. Moreover, outdoor NO_2_ concentrations and NO*_X_* emissions have been falling steadily in the United States [U.S. Environmental Protection Agency (EPA) 2013]. Nonetheless, associations of air pollutant exposures with other health outcomes, such as cardiopulmonary mortality, have been remarkably robust across space and time, and some evidence hints at the lack of an effect threshold for certain outcomes [e.g., stroke (Stafoggia et al. 2014; Wellenius et al. 2012b), lung cancer mortality (Pope et al. 2011), and total mortality (Correia et al. 2013)] or an exposure–response association that is steeper at lower levels of exposure for other outcomes [e.g., cardiovascular mortality (Pope et al. 2011)]. Moreover, ambient pollutant concentrations in some regions of the world far exceed those in the CHAP study area, and effect modification notwithstanding, our results may be pertinent to populations in those areas.

Finally, unlike long-term NO*_X_* exposure, road proximity was not consistently associated with progression in physical disability. Distance to road has been associated with several adverse health outcomes, notably incident cardiovascular disease and mortality (Adar and Kaufman 2007), but other studies that have detected associations between ambient pollutant concentrations and health outcomes have not detected comparable associations for residential distance to road [e.g., (Brunekreef et al. 2009)]. In the context of CHAP, data from thousands more participants and several more years of follow-up were available for the analysis of road proximity than for NO*_X_* exposure, but it is possible that any increase in statistical power offered by the road proximity measures was offset by their coarseness. For example, unlike NO*_X_* estimates, these measures do not account for traffic counts or differentiate between exposures emanating from a single road and exposures from two or more nearby roads. In addition to being conducted in different populations that were born approximately a decade apart, the primary analyses of distance to road and those of NO*_X_* exposure entailed exposures assessed in different periods (as early as 1993 vs. 1999–2004 for most) and outcome data beginning at different time points (as early as 1993 vs. 2004). When we restricted the analysis of A3 roadway proximity to the 5,708 participants and 10,911 observations from the NO*_X_* analyses, progressively closer proximity was associated with significantly worse progression in disability (see Table S1), consistent with the corresponding NO*_X_* association and supporting the possibility that the populations used in the primary analyses and/or their corresponding measurements may have differed in substantial ways.

## Conclusion

In conclusion, higher long-term exposure to ambient NO*_X_* was associated with a greater decline in physical function among older adults living in Chicago, Illinois. This association was detected in a population with TRAP exposures consistent with current levels in some areas of the United States and in many areas worldwide. Therefore, if our findings are confirmed by other studies, air pollution reduction may be a means for reducing the population burden of physical disability and dependence.

## Supplemental Material

(355 KB) PDF
