# Supplementary material for: Exposure to Traffic-Related Air Pollution in Relation to Progression in Physical Disability among Older Adults
Source: Environ Health Perspect. 2016 Mar 29;124(7):1000–8. doi: 10.1289/ehp.1510089 (PMC4937863; doi:10.1289/ehp.1510089)
Supplement: (355 KB) PDF [file ehp.1510089.s001.acco.pdf]

Environ Health Perspect
DOI: 10.1289/ehp.1510089

Note to readers with disabilities: *EHP* strives to ensure that all journal content is accessible to all readers. However, some figures and Supplemental Material published in *EHP* articles may not conform to 508 standards due to the complexity of the information being presented. If you need assistance accessing journal content, please contact ehp508@niehs.nih.gov. Our staff will work with you to assess and meet your accessibility needs within 3 working days.

Supplemental Material

Exposure to Traffic-Related Air Pollution in Relation to Progression in Physical Disability among Older Adults

Jennifer Weuve, Joel D. Kaufman, Adam A. Szpiro, Cynthia Curl, Robin C. Puett, Todd Beck, Denis A. Evans, and Carlos F. Mendes de Leon

Table of Contents

Computation of area-level socioeconomic status

Table S1. Adjusted differences (and 95% confidence interval [CI]) in 5-year change in physical performance by exposure to traffic-related air pollution: results from additional sensitivity analyses.

Table S2. Distribution of baseline residential distances to major road.

Table S3. Participant characteristics by category of distance to census class A3 road at baseline

Figure S1. Differences in baseline and 5-year change in physical performance score with higher long-term NO_X exposure: results with and without multivariable adjustment and weighting.

Figure S2. Model-based estimated trajectory in physical performance, by quartile of long-term exposure to ambient oxides of nitrogen (NO_X)

Figure S3. Adjusted difference (95% confidence interval [CI]) in baseline physical performance score, by residential distance to census feature class A3 road (N=9994)

Computation of area-level socioeconomic status.

We obtained the following variables for each U.S. census block group located within the CHAP region: median household income; median home value; percentage of households with income from interest, dividends or rent; percentage of adults who completed college; and the percentage of employed persons 16 years of age or older in executive, managerial, or professional specialty occupations. Following a previously developed approach (Diez Roux et al. 2001), we computed a composite area-based SES score for each block group. This score was the average of the block group-specific *z*-scores of all of the components listed above. (In a departure from the previously developed score, our score did not include percentage of adults with a high school diploma, because, in the CHAP area, this variable was not distributed as expected across composite scores.) Higher scores reflected less socioeconomic disadvantage.

Reference

Diez Roux AV, Merkin SS, Arnett D, Chambless L, Massing M, Nieto FJ, et al. 2001. Neighborhood of residence and incidence of coronary heart disease. N Engl J Med 345(2):99-106.

Table S1. Adjusted[a] differences (and 95% confidence interval [CI]) in 5-year change in physical performance by exposure to traffic-related air pollution: results from additional sensitivity analyses.

	Difference in 5-year change in physical performance score (95% CI)
Quartile of long-term[b] NO_X exposure (N=5708)	
Using truncated weights[c]	
Lowest (20.7-36.2 ppb)	0.00 *reference*
2nd (36.3-39.6 ppb)	-0.63 (-1.12, -0.14)
3rd (39.7-43.7 ppb)	-0.60 (-1.15, -0.05)
Highest (43.8-56.0 ppb)	-1.01 (-1.60, -0.42)
	p_{trend}[d] = 0.001
Unweighted	
Lowest (20.7-36.2 ppb)	0.00 *reference*
2nd (36.3-39.6 ppb)	-0.54 (-0.88, -0.19)
3rd (39.7-43.7 ppb)	-0.41 (-0.80, -0.01)
Highest (43.8-56.0 ppb)	-0.57 (-1.00, -0.14)
	p_{trend}[d] = 0.02
Residential distance to road (N=9994)	
Distance to nearest A3 road, alternative categories	
$\geq$ 500 m	0.00 *reference*
250-499 m	0.14 (-0.06, 0.34)
100-249 m	-0.05 (-0.25, 0.15)
< 100 m	0.03 (-0.19, 0.25)
	p_{trend}[d] = 0.4
Near busy road[e], yes vs no	-0.03 (-0.19, 0.13)
Residential distance to road restricted to participants in analyses of long-term[b] NO_X exposure (N=5708)	
Residential distance to nearest A3 road[f]	
$\geq$ 200 m	0.00 *reference*
100-199 m	-0.08 (-0.35, 0.19)
50-99 m	-0.17 (-0.54, 0.20)
< 50 m	-0.42 (-0.81, -0.03)
	p_{trend}[d] = 0.05

[a] Adjusted for age, sex, race, education, income, and smoking status.
[b] Predicted exposures averaged over 5 years.
[c] Extreme weights truncated to the lowest 0.5 percentile and highest 99.5 percentile.
[d] Trend p-values were computed from models containing a term that took on the median values of each category.
[e] < 100 m from truck route or A1 or A2 road, or < 50 m from A3 road.
[f] At the start of the 5-year NO_X averaging interval (1999 for most participants).

Table S2. Distribution of baseline residential distances to major road.

	Number	Percentage
Distance to Class A1 road		
Closer than 50 m	58	0.6%
50 m - 99 m	196	2%
100 m - 199 m	492	5%
200 m or farther	9248	93%
Distance to Class A2 road		
Closer than 50 m	97	1%
50 m - 99 m	356	4%
100 m - 199 m	794	8%
200 m or farther	8747	88%
Distance to Class A3 road		
Closer than 50 m	938	9%
50 m - 99 m	1091	11%
100 m - 199 m	2553	26%
200 m or farther	5412	54%
Distance to truck route		
Closer than 50 m	50	0.5%
50 m - 99 m	163	2%
100 m - 199 m	496	5%
200 m or farther	9285	93%
Minimum distance to Class A1 or A2 road		
Closer than 50 m	155	2%
50 m - 99 m	537	5%
100 m - 199 m	1203	12%
200 m or farther	8099	81%
Near busy road (< 100 m from A1, A2 or truck route; or < 50 m from A3)		
No	8414	84%
Yes	1580	16%

Table S3. Participant[a] characteristics[b] by category of distance to census class A3 road at baseline.

		Residential distance to class A3 road				
	Overall[c]	Closer than 50 m	50-99 m	100-199 m	200 m or farther	
	N=9994	N=938	N=1091	N=2553	N=5412	***P* value**
Age, years; mean (sd)	74.1 (7.1)	74.1 (7.0)	73.7 (6.8)	75.0 (7.8)	73.7 (6.8)	< 0.0001
Male, %	3885 (39)	34	38	38	40	0.005
African American, %	6296 (63)	73	70	61	61	< 0.0001
Education, years; mean (sd)	12.3 (3.5)	11.5 (3.5)	11.9 (3.4)	12.2 (3.4)	12.6 (3.5)	< 0.0001
Household income, %[d]						
< $15,000	2809 (28)	36	31	29	26	
$15,000-$29,999	3471 (35)	38	37	35	34	< 0.0001
> $30,000	3266 (33)	22	28	31	36	
Missing	448 (4)	4	4	5	4	
Self-rated health, %[d,e]						
Excellent	2217 (22)	20	21	20	24	
Good	4715 (47)	46	45	49	47	< 0.0001
Fair	2509 (25)	27	29	25	24	
Poor	542 (5)	8	5	6	5	
Global cognitive score, standard units; mean (sd)	0.2 (0.8)	0.1 (0.8)	0.1 (0.8)	0.1 (0.8)	0.3 (0.8)	0.0002
Social network score, median (iqr)[e]	6 (7)	6 (7)	6 (7)	5 (6)	6 (7)	< 0.0001
Walks > 3 h per week, %[e]	2243 (23)	19	22	23	23	0.09
Smoking status, %[d]						
Never	4596 (46)	46	46	48	45	
Former	4027 (40)	39	38	38	42	0.007
Current	1371 (14)	15	16	13	13	
Alcohol intake, %[d,e]						
None	6844 (69)	74	71	70	66	
Moderate	2464 (25)	20	22	24	26	< 0.0001
Heavy	671 (7)	6	6	6	7	
Systolic blood pressure, mm Hg; mean (sd)[e]	137 (20)	137 (20)	138 (21)	137 (20)	138 (20)	0.7
Diastolic blood pressure, mm Hg; mean (sd)[e]	78 (12)	78 (12)	79 (12)	78 (11)	78 (11)	0.07
Self-reported history of:						
Hypertension, %[e]	5482 (55)	54	58	55	55	0.3
Cardiovascular disease, %[e]	1420 (14)	15	15	14	14	0.7
Stroke, %[e]	997 (10)	12	9	10	9	0.05
Cancer, %[e]	1972 (20)	18	21	20	20	0.3
Moved over the course of follow-up, %	20	25	20	20	20	0.002
Physical performance score: mean (sd)	9.8 (3.9)	9.2 (4.0)	9.7 (3.8)	9.5 (4.0)	10.0 (3.9)	< 0.0001

[a] Among participants included in the analyses of distance to road and physical performance, among whom baseline was defined as the first physical performance assessment on or after study enrollment (1993-2008).
[b] Unless specified, all characteristics reflect baseline values, i.e., the values at the first physical performance assessment on or after study enrollment (1993-2008).
[c] Where percentages are listed in the "Overall" column, counts are also provided [n (%)].
[d] Some columns do not sum to 100 percent due to rounding.
[e] Data on self-rated health, social network score, walking, alcohol intake, blood pressure, and self-reported history of the four chronic conditions were missing for a small percentage of participants (<4%). Values shown reflect non-missing responses. Data on these characteristics were not used in the association models.

Figure S1. Differences in baseline and 5-year change in physical performance score with higher long-term[a] NO_X exposure: results with and without multivariable adjustment[b] and weighting[c].

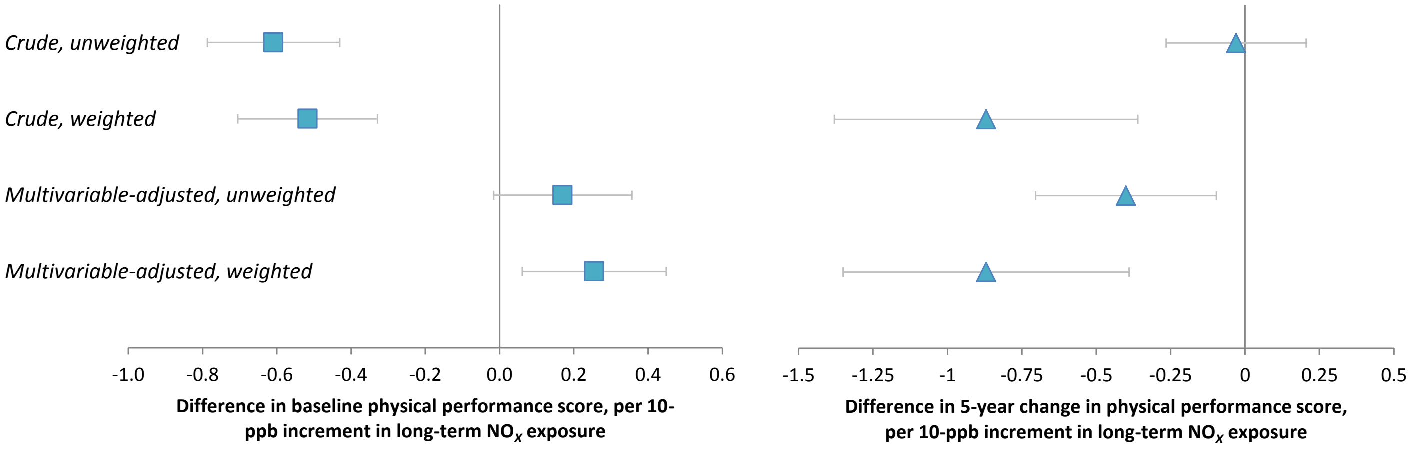

[a] Predicted exposures averaged over 5 years, unless another interval is specified.

[b] Adjusted, via independent terms in the regression models, for age, sex, race, education, income, and smoking status.

[c] Application of inverse probability-of-continuation weights.

Figure S2. Model-based estimated trajectory in physical performance[a], by quartile of long-term[b] exposure to ambient oxides of nitrogen (NO_x).

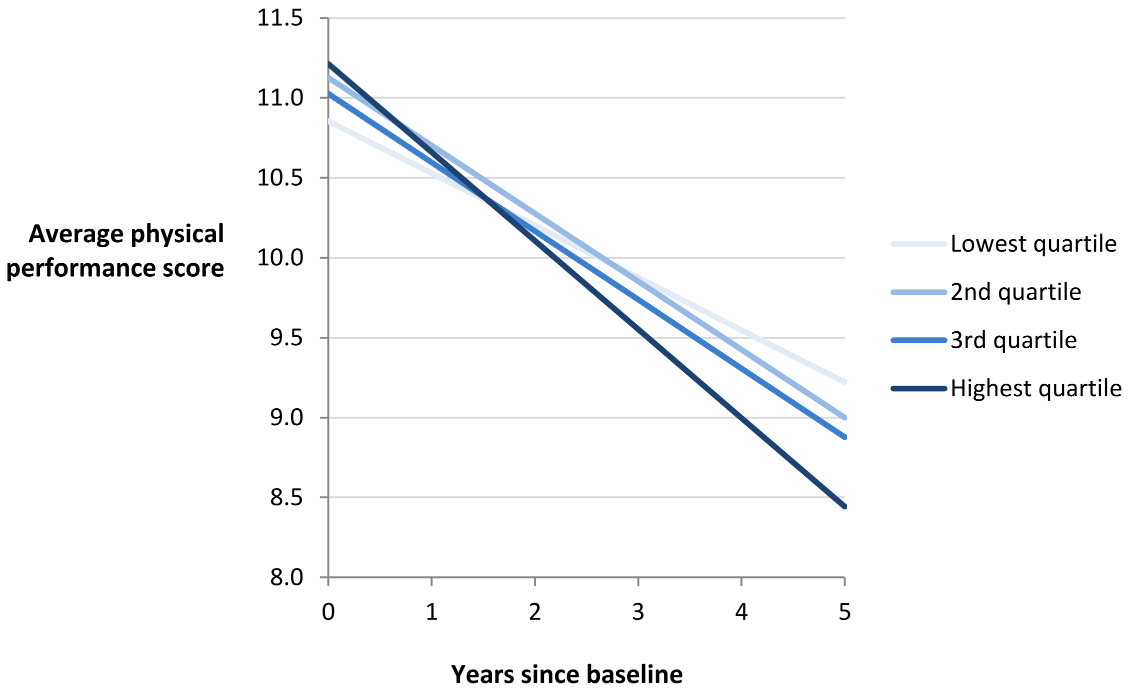

[a] Estimated physical performance score based on weighted generalized estimating equations regression model. The model was adjusted for age, sex,race, education, income, and smoking status.
[b] Predicted exposures averaged over 5 years prior to baseline physical performance.

Figure S3. Adjusted[a] difference (95% confidence interval [CI]) in baseline physical performance score, by residential distance to census feature class A3 road (N=9994).

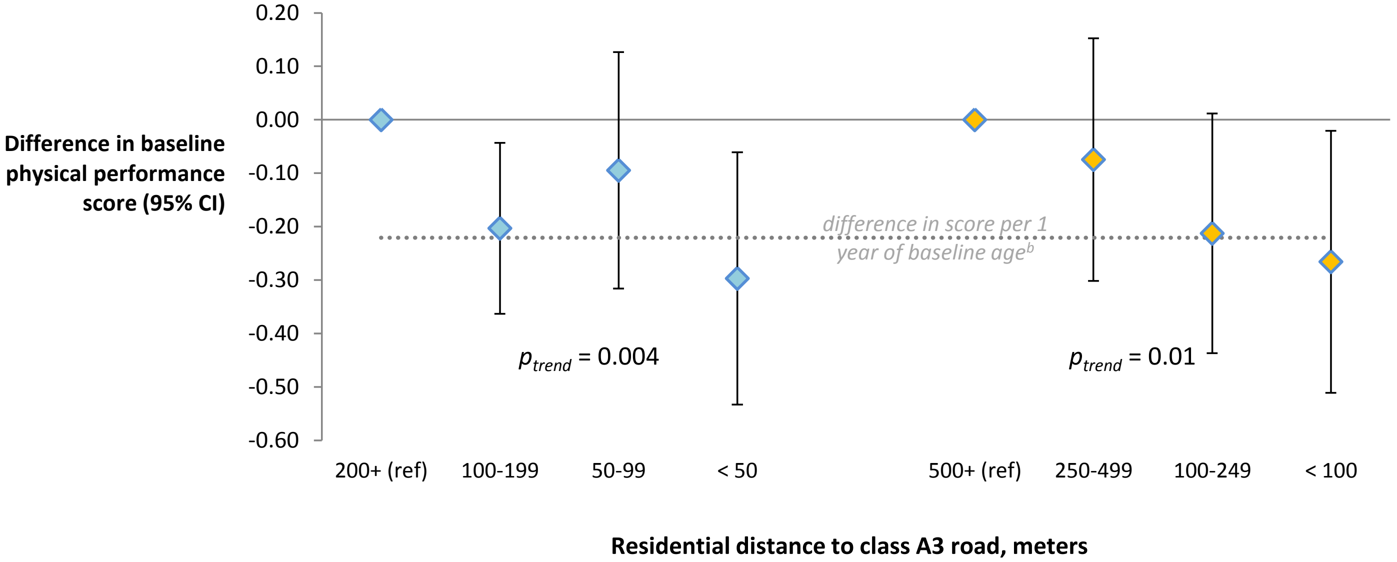

[a] Adjusted for age, sex, race, education, income, and smoking status. [b] For context on magnitude of the differences, the difference per one-year older age at baseline was -0.22 units in physical performance score.
